# The application potential of mepiquat chloride in soybean: improvement of yield characteristics and drought resistance

**DOI:** 10.1186/s12870-024-05028-1

**Published:** 2024-04-23

**Authors:** Xiyue Wang, Wei Zhao, Xinhe Wei, Shuang Song, Shoukun Dong

**Affiliations:** College of Agriculture, Northeast Agricultural University, Harbin, 150030 China

**Keywords:** *Glycine max*, Mepiquat chloride, Drought regulation, Yield improvement, Plant phenotypic regulation, Antioxidant system

## Abstract

**Background:**

Drought can result in yield losses, the application of plant growth regulators is an effective measure to improve drought resistance and yield. The objective of the study was to explore the application potential of mepiquat chloride (MC) in regulating soybean yield and drought resistance.

**Methods:**

In this study, a three-year field experiment was designed and combined with drought experiments to measure the yield of popularized varieties during 2021–2022 and drought-resistant and drought-sensitive varieties were selected, and planted in the field in 2023.

**Results:**

MC increased the yield of HN84 and HN87 for two consecutive years from 2021 to 2022 and improved their physiological characteristics under field conditions. Under M200 treatment, the yield of HN84 increased by 6.93% and 9.46%, and HN87 increased by 11.11% and 15.72%. Different concentrations of MC have different effects on soybeans. The maximum increase of SOD, POD and proline in HN84 under M400 treatment reached 71.92%, 63.26% and 71.54%, respectively; the maximum increase of SOD, POD and proline in HN87 under M200 treatment reached 21.96%, 93.49% and 40.45%, respectively. In 2023, the foliar application of MC improved the physiological characteristics of HN44 and HN65 under drought-stress conditions. On the eighth day of drought treatment, compared to the drought treatment, the leaf and root dry weight of HN44 under M100 treatment increased by 17.91% and 32.76%, respectively; the dry weight of leaves and roots of HN65 increased by 20.74% and 29.29% under M200 treatment, respectively. MC also reduced malondialdehyde (MDA) content, decreased antioxidant enzyme activity and proline content. In addition, different concentrations of MC increased the chlorophyll fluorescence parameters (Fs, Fv/Fm, YII, and SPAD). In the field, the plant height of the two varieties decreased significantly, the yield increased, the number of two-grain and three-grain pods increased, and the stem length at the bottom and middle decreased with MC induction.

**Conclusions:**

The application of 100–200 mg/L MC effectively improved drought resistance and increased yield. This study provided support for the rational application of MC in soybean production.

**Supplementary Information:**

The online version contains supplementary material available at 10.1186/s12870-024-05028-1.

## Introduction

Soybean (*Glycine max*) was originally defined as an important food and oil crop and is a major source of plant proteins and oils [1]. With the improvement of technology and the development of new varieties, soybeans have been widely used in animal husbandry, industry, and medical industries, and global soybean production and yield per hectare have increased steadily [2]. However, the current global soybean supply and demand remain uneven. In China, there is a serious shortage of soybean supply, and the import dependence has exceeded 80%, which may lead to food security risks [3]. In addition, the intensity of global climate change has recently increased, and variations in the frequency and intensity of floods and droughts can seriously affect farmers and jeopardize food safety [4]. Therefore, soybean yield needs to be further improved.

In soybean production, many major factors lead to yield losses annually. Different environmental conditions are often subjected to biotic or abiotic stresses, which are the main factors affecting soybean production [5]. Drought is one of the most serious abiotic stresses, which causes more than 10% of the global soybean yield loss in each harvest period [6]. Gebre et al. [7] reported that drought stress decreased soybean seed yield by 51% on average. Drought resistance and yield of soybeans can be improved through genetic engineering. Chen et al. [8] constructed transgenic plants overexpressing MYB14 in soybean, which produced a semi-dwarf and compact plant structure as well as increased yield under high-density planting in the field. However, the breeding of transgenic varieties is costly and also has a long breeding period.

Using contemporary agricultural technologies will help soybeans become more resilient to abiotic stress, more adaptable to a variety of conditions, and more productive given the rising demand for the crop. The growth, development, yield, quality, and abiotic stress responses of soybeans are significantly affected by plant growth regulators, which are crucial to this process [9]. Mepiquat chloride (MC) is an endogenous plant growth regulator that plays a vital role in regulating plant growth and yield. MC was first applied in cotton production and can efficiently lower plant height, leaf area, pitch, and canopy size through foliar application, improving the capacity of the canopy to intercept light and boost cotton output [10]. The application of MC at the flowering stage effectively reduced plant height and increased the seed yield of castor (*Ricinus communis* L.) plants [11]. MC can also improve plant stress resistance. The use of MC under salt stress can significantly increase dry weight, fresh weight, glycine betaine content, and proline and phenolic compounds in cotton [12]. Additionally, MC enhanced the GSH-ASA and Calvin cycles of soybeans during drought stress to lessen oxidative damage and preserve crop development [13]. Therefore, MC has considerable application potential in the regulation of crop yield and stress resistance.

Heilongjiang Province is a concentrated soybean-producing area in China, and in recent years, drought in summer and autumn has been increasing, significantly affecting the growth and development of soybeans, thereby affecting yield and quality [14]. Therefore, this study designed two experiments to explore the application potential of MC in regulating soybean yield and drought resistance. In experiment 1, the study explored the effects of MC on the yield and physiological characteristics of field crops by measuring the yield of popularized varieties over two consecutive years (2021–2022), and in experiment 2, drought-resistant and drought-sensitive varieties were selected, and planted in the field in the same year (2023) to explore the physiological mechanisms of MC regulation in soybean drought resistance. The yield was verified, and the effect of MC on the yield formation characteristics was explored. These data further confirm the application potential of MC in soybean production and provide support for the rational application of MC in crop production.

## Materials and methods

### Overview of field experiments

The field experiment (2021–2023) was carried out at the Xiangyang Experimental Base of Northeast Agricultural University (Heilongjiang Province, China)(126°91′ E, 45°77′ N). The soil type is black soil, and the basic soil fertility is shown in Table S1. The cultivation method adopted the strip deep-loosening cultivation technique of straw mulching and returning to the field. The previous corn was harvested, the straw was crushed, returned to the field, and evenly distributed. After crushing, the straw length was ≤ 10 cm, and the stubble height was ≤ 10 cm. A straw ridge-furrow subsoiling stubble preparation machine (model: 1SMZ-130) was used. The depth of subsoiling was 30 cm, the width of the clean stubble was 35 cm, the depth of the clean stubble was 12 cm, and the soil was fine and loose. After mechanical ditching, diammonium phosphate 10 kg/mu (P_2_O_5_: 46%) and potassium sulfate 5 kg/mu (K_2_O: 52%) fertilizers were applied. The 2MMZ-2 no-tillage fertilization planter developed by Northeast Agricultural University was used to complete fertilization at one time, and then manually sown. Two grains were placed in each position, and plant spacing was 12 cm, ridge spacing was 65 cm, seedling spacing was performed after full seedling, and seedling density was 250,000 plants/ha. When soybean grew to the 2–3 leaf stage, the furrow was deeply loosened to a depth of 25 cm, and the soil was plowed and cultivated once during the initial flowering period.

### Experimental design 1 in 2021–2022

This study used Heinong 84 (HN84) and Heinong 87 (HN87) varieties, which were approved and promoted by the Heilongjiang Province in 2017. A randomized block design was used in this study. Each treatment consisted of three replicate plots, and each plot had eight ridges with a ridge length of 5.0 m and a ridge width of 0.65 m. An electric sprayer was used to spray the MC solution on the leaves of the plant at the end of soybean vegetative growth (before the initial flowering stage) and was sprayed again after seven days, a total of two times. The MC concentrations in the treatment groups were set to 100, 200, and 400 mg/L, respectively, and the control group (CK) was sprayed with water on the leaves. During the harvest period (R8), each treatment was repeated three times for yield measurements.

In the 2022 experiment, the SPAD values of the leaves were measured at R2 (full flowering stage), R3 (initial pod stage), R4 (full pod stage), R5 (initial grain stage), and R6 (seed-filling stage), and the leaves of each treatment were sampled simultaneously. The sampling sites were the second and third leaves near the top. The samples were then placed in an ultralow-temperature refrigerator for testing.

### Experimental design 2 in 2023

The experimental varieties used were Heinong 44 (HN44: drought-resistant) and Heinong 65 (HN65: drought-sensitive) [15]. The MC concentration was further increased, and the MC concentration in the treatment group was set to 50, 100, 200, 400, and 800 mg/L, respectively. The other experimental conditions were the same as those in previous years, and the yield and yield characteristics were evaluated at the harvest stage.

### Drought simulation experiment design

The following tests were conducted to investigate the regulatory role of MC in drought stress conditions. In 2023, the experiment was conducted at the Northeast Agricultural University Experimental Station (126°72′ E, 45°74′ N). The experimental varieties used in this study were Heinong 44 and Heinong 65 [15]. Eight grains were sown in each pot using the sand culture pot method. Three seedlings were maintained in each pot after the seedlings were divided after the true leaves had fully developed. A suitable water supplement was added before seeding until the opposite true leaves were fully developed. Once the true leaves were expanded, 500 mL of modified Hoagland nutrition solution was added daily. The treatment began when the soybeans grew to the three-leaf stage (V3 stage). Treatment 1 (normal water condition, CK): Normal water conditions were maintained; that is, 500 mL of modified Hoagland nutrition solution was poured once a day. Treatment 2 (drought treatment, DS): After three days of maintaining typical water conditions, the drought stress treatment was applied. To replicate drought stress, 500 mL of modified Hoagland nutrient solution containing 15% PEG-6000 was added once a day for a total of four samplings every two days. Treatment 3–6 (drought + MC treatment): All leaves were sprayed with MC solution (50, 100, 200, and 400 mg/L) until they were thoroughly moist without dripping. Drought stress was introduced after three days, and 500 mL of nutrient solution containing 15% PEG-6000 was added once daily. Thereafter, sampling was performed twice daily for four times. At least three biological replicates were used for each treatment.

### Determination of morphological and physiological indices

The plant height was determined using a meter ruler to measure the cotyledon scar to the top of the main stem growth point.

Determination of dry weight: During each sampling, three pots of soybean plants with uniform growth were selected for each treatment. The leaves, stems, and roots of the plants were manually separated, and the roots were washed with clean water. Subsequently, each part was placed in a kraft paper bag and then in the oven. First, it was deactivated at 105 °C for 30 min, dried at 65 °C to a constant weight, and the dry weight was measured using a balance.

Physiological indicators were measured using kits (Norminkoda Biotechnology Co., Ltd. Wuhan, China). The NBT method was used to measure SOD, the guaiacol colorimetric approach was used to detect POD, UV colorimetric method was used to measure CAT activity, sulfosalicylic acid-ninhydrin method was used to measure proline content, and thiobarbituric acid colorimetric method was used to measure MDA levels.

Determination of chlorophyll fluorescence characteristics: steady-state fluorescence yield (Fs), pSII photochemical quantum efficiency (Fv/Fm), actual light energy conversion efficiency (YII), and relative chlorophyll (SPAD) of soybeans were measured using a multifunctional plant-measuring instrument (Model: MultispeQV2.0). Each test was repeated at least six times.

Determination of yield and yield characteristics: During R8, 2 m^2^ was sampled from each plot, and each treatment was repeated three times. For each replicate, 15 plants were randomly selected for the determination of yield characteristics. After threshing, the 100-grain weight and yield per mu of soybeans were measured.

### Statistical analysis

Physiological figures and tables were drawn using Microsoft Office Excel 2021 (USA), and the values are the mean (*n* ≥ 3) ± standard error (SEs). One-way analysis of variance (ANOVA) was performed using IBM SPSS Statistics 26 software to determine significant differences between treatments.

## Results

### MC has a promoting effect on yield

The MC treatment increased the 100-grain weight of the soybeans (Table 1). In 2021, the 100-grain weight of HN84 increased slightly, but the difference between the treatments was not significant. In 2022, M200 and M400 treatments were significantly higher than CK, with an increase of 4.92% and 8.56%, respectively. For the HN87 variety, the 100-grain weights for each treatment in 2021 were significantly different. Compared with CK, M100, M200, and M400 increased by 13.60%, 5.45%, and 6.70%, respectively. In 2022, no significant difference was observed in the 100-grain weight among the treatments.

**Table 1:** Effects of MC on 100-seed weight and yield of soybean

Varieties	Year	Treatment	100-seed Weight(g)	Yield(kg/mu)
HN84	2021	CK	20.2 ± 0.05a	203.88 ± 2.64bc
		M100	20.3 ± 0.52a	208.03 ± 1.09b
		M200	20.5 ± 0.70a	223.17 ± 4.02a
		M400	20.6 ± 0.12a	199.09 ± 6.01c
	2022	CK	20.33 ± 0.23c	203.99 ± 1.08d
		M100	20.97 ± 0.27bc	215.03 ± 0.91b
		M200	21.33 ± 0.38ab	218.13 ± 1.04a
		M400	22.07 ± 0.12a	208.01 ± 0.63c
HN87	2021	CK	15.96 ± 0.21c	168.10 ± 3.71c
		M100	18.13 ± 0.15a	209.43 ± 1.86a
		M200	16.83 ± 0.25b	186.78 ± 5.07b
		M400	17.03 ± 0.20b	187.16 ± 15.72b
	2022	CK	18.44 ± 0.03a	188.51 ± 0.70c
		M100	18.52 ± 0.06a	191.85 ± 0.91b
		M200	18.67 ± 0.15a	218.14 ± 1.09a
		M400	18.42 ± 0.04a	191.25 ± 0.90bc

*Note* In the same index and the same year of the same variety, there is a significant difference at the 5% level between different letters representing treatments (*P* < 0.05)

The application of MC had a potential yield-increasing effect (Table 1). In 2021, the yields (HN84) of M100 and M200 were higher than those of the CK, whereas the yield of M400 decreased. In 2022, the yield of each MC treatment increased, and the M200 treatment showed the best yield increase effect in two consecutive years, with an increase of 9.46% and 6.93%, respectively. In the HN87 variety, each MC treatment resulted in an increase in yield over two consecutive years. In 2021, each treatment increased by 24.59%, 11.11%, and 11.34%, respectively, compared with CK. In 2022, each treatment increased by 1.77%, 15.72%, and 1.45%, respectively, compared to CK.

### MC improved the physiological characteristics of field soybeans

**Fig. 1:**
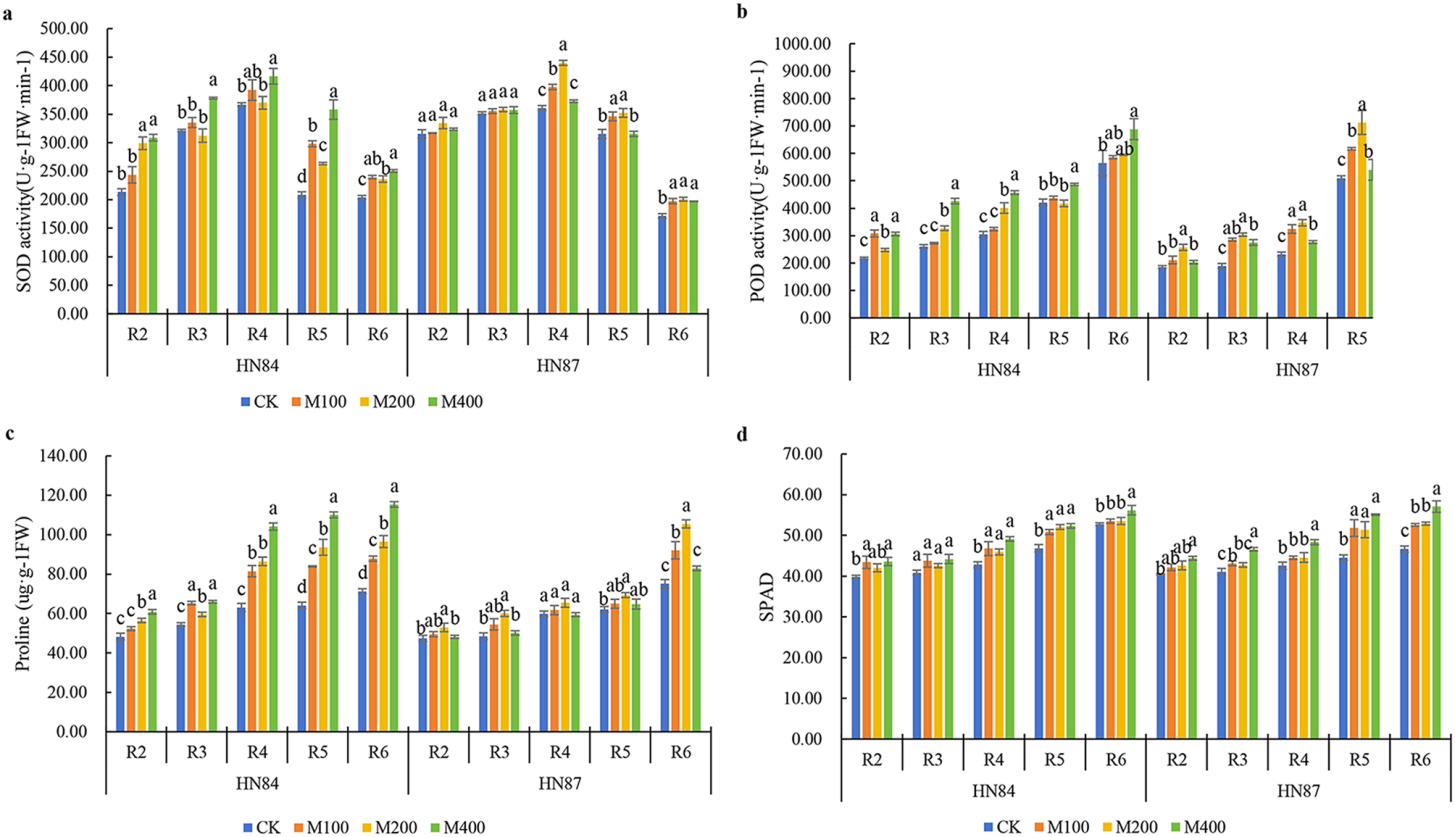
Effects of MC on physiological characteristics of flowering soybean. **a**, SOD activity; **b**, POD activity; **c**, proline content; **d**, SPAD index. *Note* In the same period of the same variety, different letters indicate significant differences between treatments (*P*<0.05)

MC altered the physiological characteristics of plants at different growth stages. From the R2 to R6 stages, the SOD activity of the leaves of the two varieties first increased and then decreased after treatment with MC (Fig. 1a). The MC treatment of HN84 reached its peak at the R4 stage and had a significant improvement effect at a concentration of 400 mg/L. From R2 to R6, HN84 increased by 44.82%, 17.66%, 13.50%, 71.92%, and 22.83%, respectively, compared with the control. The MC treatment of HN87 reached a peak at R4 and had a significant improvement effect at a concentration of 200 mg/L. From R2 to R6, HN87 increased by 5.94%, 1.90%, 21.96%, 11.69%, and 17.13%, respectively, compared with the control. HN84 and HN87 had the best promotional effect in the R5 and R4 stages, respectively.

With the advancement of the growth process, the POD activity of the leaves of the two varieties under the MC treatment showed a gradually increasing trend (Fig. 1b). The MC treatment of HN84 reached its peak at the R6 stage, and the effect was most significant at a concentration of 400 mg/L. From R2 to R6, HN84 increased by 40.81%, 63.26%, 50.19%, 15.56%, and 21.94%, respectively, compared with the control. HN87 had a significant improvement effect at a concentration of 200 mg/L. From R2 to R6, HN87 increased by 38.65%, 60.35%, 49.11%, 39.69%, and 93.49%, respectively, compared to the control at a concentration of 200 mg/L MC. In addition, HN84 in R3 and HN87 in R6 showed the best effects.

The application of MC promoted an increase in the free proline content of leaves under field conditions. From R2 to R6, the proline content of the two varieties showed a gradual upward trend under the MC treatment (Fig. 1c). The MC treatment of HN84 reached its peak at the R6 period and had a significantly increasing effect at a concentration of 400 mg/L. From R2 to R6, HN84 increased by 25.97%, 21.28%, 64.99%, 71.54%, and 61.82%, respectively, compared with the control. HN87 had a significant increase effect at a concentration of 200 mg/L. From R2 to R6, HN87 increased by 11.71%, 23.92%, 9.30%, 11.73%, and 40.45%, respectively, compared to the control at a concentration of 200 mg/L MC. HN84 and HN87 showed the best effects in the R5 and R6 stages, respectively.

The application of MC promoted an increase in the relative chlorophyll content of leaves under field conditions (Fig. 1d). Over time, the SPAD of the two soybean varieties gradually increased. The SPAD values of the leaves of the HN84 variety reached a maximum in the M400 treatment at each period, which increased by 9.49%, 8.20%, 14.74%, 11.70%, and 6.54%, respectively, compared with the control at each period. In the R6 period, the M400 treatment was significantly higher than the M100 and M200 treatments. In the HN87 variety, the M400 treatment increased SPAD by 9.71%, 13.46%, 13.53%, 23.66%, and 22.41%, respectively, compared with the control. In the sprayed MC treatment, except for the R5 and R2 period, M400 was significantly higher than other treatments. In summary, MC effectively increased the SPAD value of leaves. HN84 and HN87 had the best effect at a concentration of 400 mg/L.

### Effects of MC on plant height and yield characteristics

**Fig. 2:**
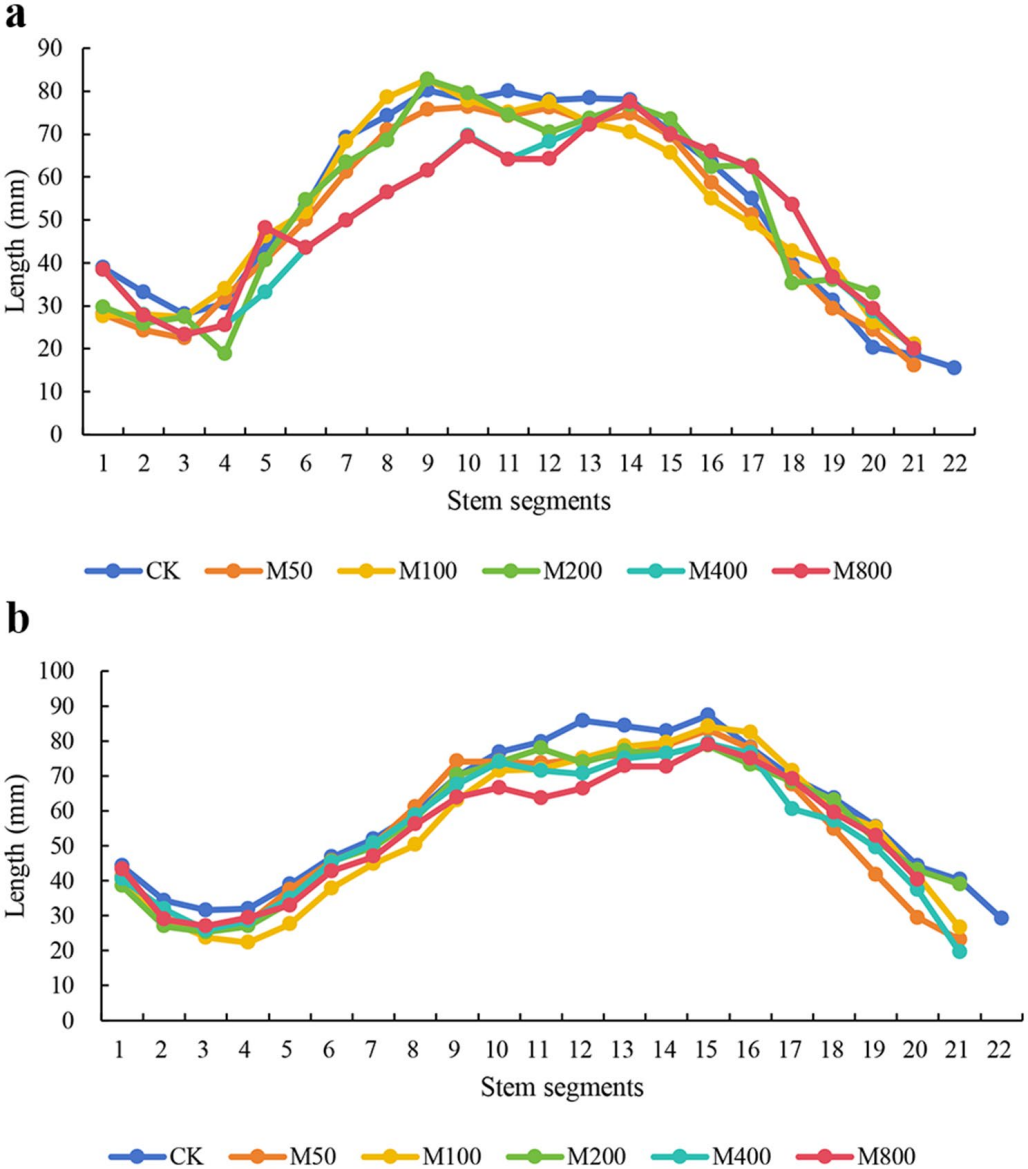
The length of each stem node under each treatment. **a**, HN44; **b**, HN65. *Note* The data in the figure come from the average of at least 15 samples (*n* ≥ 15), so the figure does not mark the error line and the significant difference

Application of MC in the field resulted in a decrease in plant height. With increasing concentrations, the inhibitory effect on plant height became more pronounced. Except for the M50 treatment in HN44, all treatments were significantly different from CK. When the degree of inhibition was the greatest, the plant height of HN44 decreased by 10.16% (M200), and the plant height of HN65 decreased by 15.92% (M800 treatment). The number of internodes showed a downward trend in the two varieties, and some concentrations were significantly different from those in the CK (Table 2). We further counted the changes in the length of each internode (1–22 internodes) between the two varieties at each MC concentration. Notably, in HN44, the lengths of the 1–3 and 10–15 internodes in the CK treatment were greater than those in each MC treatment. At HN65, the 1–7 and 10–15 internodes in the CK treatment were higher than those in the MC treatments (Fig. 2). These results suggest that MC may reduce plant height by reducing the length of the bottom and middle internodes.

**Table 2:** Effects of MC on plant height and internode number

Varieties	Treatments	Plant height(cm)	Internode number
HN44	CK	105.64 ± 0.89a	19.71 ± 0.29a
	M50	101.62 ± 1.36ab	18.93 ± 0.32abc
	M100	99.41 ± 1.59bc	18.20 ± 0.39c
	M200	94.91 ± 2.17c	18.80 ± 0.22abc
	M400	95.12 ± 1.18c	18.67 ± 0.23bc
	M800	95.23 ± 1.46c	19.40 ± 0.41ab
HN65	CK	118.31 ± 1.14a	20.67 ± 0.27a
	M50	113.29 ± 1.24b	19.60 ± 0.29b
	M100	108.35 ± 1.32c	20.00 ± 0.32ab
	M200	107.71 ± 1.05c	19.53 ± 0.32b
	M400	104.85 ± 1.51c	19.07 ± 0.33bc
	M800	99.47 ± 1.07d	18.40 ± 0.35c

*Note* Refer to Table 1 for descriptions to Tables

Table 3 shows the field yield characteristics of the drought-resistant and drought-sensitive varieties. In the drought-resistant variety HN44, no significant difference was observed in the number of pods between the treatments. Compared with CK, with an increase in the application concentration, the number of one- and two-seed pods increased or decreased, and an inverse relationship was observed between one- and two-seed pods. The number of three-seed pods was higher than that of CK at each concentration, and the M100 treatment reached the highest, which was 10.70% higher than that of CK. The number of four-seed pods was lower than that of the CK in each MC treatment. The M50 treatment significantly increased the 100-grain weight, and there was no significant difference between the other treatments and the CK, which increased or decreased slightly. In terms of yield, each treatment increased the yield. A significant difference was observed between the M400 treatment and CK, and the yield increased by 7.13–11.5% among the treatments.

In the sensitive variety HN65, a significant difference was observed in the number of pods between treatments. Compared with the CK, the number of one seed pods decreased at each MC concentration, especially at low concentrations (M50 and M100). The number of two- and three-seed pods increased at all MC concentrations. The number of two-seed pods increased by 39.55% at the M800 concentration, and the number of three-seed pods reached a maximum at the M100 concentration (an increase of 18.75%). The number of four-seed pods increased at a lower concentration (50–100 mg/L), and the number was lower than that in the CK, with a further increase in the concentration. There was no significant difference in the 100-grain weight between treatments. Except for the M200 treatment, the 100-grain weight of other treatments increased, and the 100-grain weight under the M100 treatment reached a maximum, which was 3.39% higher than that of CK. In terms of yield, each treatment increased the yield, but the difference between the treatments was not significant, and the yield increase between the treatments ranged from 2.32 to 5.39%.

**Table 3:** Yield characteristics among different varieties

Varieties	Treatments	Number of one-seed pods	Number of two-seed pods	Number of three-seed pods	Number of four-seed pods	100-grain weight (g)	Yield (Kg/mu)
HN44	CK	4.68 ± 0.54a	17.91 ± 1.60a	28.39 ± 1.67a	0.86 ± 0.22a	19.27 ± 0.41b	228.21 ± 0.74b
	50	5.00 ± 0.61a	16.63 ± 0.96a	29.82 ± 1.15a	0.69 ± 0.17a	20.73 ± 0.09a	249.66 ± 6.54ab
	100	4.43 ± 0.55a	18.25 ± 1.12a	31.43 ± 1.25a	0.64 ± 0.19a	18.97 ± 0.22b	245.58 ± 3.90ab
	200	5.43 ± 0.45a	16.43 ± 0.90a	28.64 ± 1.46a	0.71 ± 0.20a	19.13 ± 0.37b	248.02 ± 7.23ab
	400	5.39 ± 0.41a	16.89 ± 1.65a	28.94 ± 1.70a	0.44 ± 0.16a	18.73 ± 0.49b	254.46 ± 2.32a
	800	4.86 ± 0.53a	16.71 ± 0.89a	28.93 ± 1.05a	0.42 ± 0.13a	19.23 ± 0.19b	244.49 ± 9.13ab
HN65	CK	3.11 ± 0.25a	7.08 ± 0.46c	16.07 ± 0.74c	9.52 ± 0.61ab	18.6 ± 0.40a	218.20 ± 2.65a
	50	1.68 ± 0.27b	8.00 ± 0.65bc	17.29 ± 0.68abc	9.71 ± 0.71ab	18.97 ± 0.42a	226.16 ± 16.66a
	100	1.21 ± 0.30b	7.89 ± 0.65bc	19.07 ± 0.93a	11.54 ± 0.83a	19.23 ± 0.22a	223.28 ± 1.87a
	200	2.89 ± 0.4a	9.00 ± 0.72bc	18.17 ± 1.07abc	9.13 ± 0.89b	18.57 ± 0.34a	229.97 ± 12.93
	400	2.64 ± 0.42a	7.21 ± 0.56bc	16.26 ± 0.72bc	9.46 ± 0.82ab	19.00 ± 0.70a	225.08 ± 13.47a
	800	2.86 ± 0.29a	9.88 ± 0.53a	18.65 ± 0.80ab	8.11 ± 0.73b	18.87 ± 0.50a	225.11 ± 6.84a

*Note* Refer to Table 1 for descriptions to Tables

### Effects of MC on soybean morphology under drought stress

The plant height, leaf dry weight, stem dry weight, petiole dry weight, and root dry weight of each treatment increased with prolonged treatment time, and all reached a maximum on day 8, but the rate of increase was different (Table 4). Drought significantly reduced plant height in both varieties. On day 8, the plant heights of the two varieties decreased by approximately 25%. With an increase in the MC concentration, the inhibitory effect on plant height became increasingly evident. On day 8, the M400 treatment caused the plant height of HN44 and HN65 to decrease by 46.92% and 37.54%, respectively.

Drought significantly reduced the dry weights of leaves The M50, M100, and M200 treatments increased the leaf dry weights of the two varieties under drought stress. The M100 treatment of HN44 consistently maintained a high dry weight content during the test period, and the dry weight of leaves increased by 38.42%, 23.90%, 17.85%, and 17.91%, respectively. In HN65, the M200 treatment maintained high dry weight accumulation during the test period. Compared with DS, the dry weight of leaves increased by 10.30%, 9.71%, 15.12%, and 20.74%, respectively. The drought-resistant variety Heinong 44 showed an inhibitory effect at high MC concentrations, and the effect of MC on the leaf dry weight of Heinong 65 was greater than that of Heinong 44. The application of MC also increased the dry weight of the stems and petioles under drought stress. In Heinong 44, the M100 treatment showed a better effect, and the dry weight of stems and petioles increased by 49.40% and 12.55% on day 8. In Heinong 65, the improvement effect of the M200 treatment reached 21.02% and 24.59%, respectively.

MC promoted root dry matter accumulation under drought stress. In Heinong 44, all MC treatments increased root dry weight under drought stress. On day 8, the increase in the root dry weight of each treatment was in the order of M100 > M50 > M200 > M400. Compared to DS, the root dry weight of the M100 treatment increased by 8.81%, 23.77%, 26.67%, and 32.76%, respectively. In Heinong 65, the increase in root dry weight for each treatment on day 8 was in the order M200 > M100 > M400 > M50. Compared with DS, the root dry weight of the M200 treatment increased by 40.00%, 41.90%, 33.61%, and 29.29%, respectively.

**Table 4:** Effects of MC on plant dry matter weight under drought stress

Index	Varieties	Treatments	Drought stress treatment time			
			day 2	day 4	day 6	day 8
Plant height ( cm )	HN44	CK	30.41 ± 0.09a	43.27 ± 0.14a	52.00 ± 0.23a	59.34 ± 0.28a
		DS	28.19 ± 0.16b	34.55 ± 0.30b	41.99 ± 0.41b	44.40 ± 0.12b
		M50	25.57 ± 0.22c	32.29 ± 0.32c	36.65 ± 0.25c	38.51 ± 0.26c
		M100	24.17 ± 0.33d	32.69 ± 0.21c	35.58 ± 0.06d	38.26 ± 0.54c
		M200	23.14 ± 0.11e	28.33 ± 0.13d	34.50 ± 0.31e	35.60 ± 0.20d
		M400	23.46 ± 0.09e	26.33 ± 0.88e	29.63 ± 0.19f	31.50 ± 0.29e
	HN65	CK	24.00 ± 0.00a	37.99 ± 0.26a	47.41 ± 0.64a	50.43 ± 0.30a
		DS	24.81 ± 0.61a	32.27 ± 0.17b	35.77 ± 0.61b	37.60 ± 0.12b
		M50	23.90 ± 0.10a	27.26 ± 0.18c	29.35 ± 0.98d	32.72 ± 0.49e
		M100	20.67 ± 0.33b	25.48 ± 0.27d	31.98 ± 0.47c	36.08 ± 0.46c
		M200	24.60 ± 0.24a	27.07 ± 0.25c	31.74 ± 0.18c	34.13 ± 0.08d
		M400	16.97 ± 0.47c	19.77 ± 0.42e	28.13 ± 0.38d	31.50 ± 0.06f
Leaf dry weight (g/pot)	HN44	CK	4.95 ± 0.21a	5.39 ± 0.04a	7.18 ± 0.33a	8.23 ± 0.37a
		DS	3.41 ± 0.04c	4.10 ± 0.13d	5.21 ± 0.05c	5.64 ± 0.03c
		M50	4.36 ± 0.16b	4.88 ± 0.05bc	5.73 ± 0.01bc	5.94 ± 0.08c
		M100	4.72 ± 0.06ab	5.08 ± 0.10b	6.14 ± 0.34b	6.65 ± 0.14b
		M200	3.61 ± 0.08c	4.65 ± 0.06c	5.57 ± 0.07bc	5.76 ± 0.02c
		M400	2.73 ± 0.40d	2.87 ± 0.07e	4.40 ± 0.61d	5.06 ± 0.09d
	HN65	CK	4.79 ± 0.01a	5.39 ± 0.14a	6.05 ± 0.03a	7.07 ± 0.05a
		DS	3.30 ± 0.08e	4.53 ± 0.06 cd	5.16 ± 0.05e	5.40 ± 0.10e
		M50	4.29 ± 0.06b	4.78 ± 0.07bc	5.76 ± 0.05d	6.23 ± 0.12bc
		M100	4.10 ± 0.01c	5.48 ± 0.09a	5.87 ± 0.07c	6.05 ± 0.01 cd
		M200	3.64 ± 0.20d	4.97 ± 0.03b	5.94 ± 0.05b	6.52 ± 0.14b
		M400	3.54 ± 0.06d	4.43 ± 0.09d	5.58 ± 0.16d	5.72 ± 0.10de
Stem dry weight (g/pot)	HN44	CK	2.18 ± 0.09a	2.88 ± 0.06a	3.36 ± 0.19a	4.86 ± 0.11a
		DS	1.59 ± 0.17c	1.98 ± 0.07e	2.22 ± 0.04c	2.49 ± 0.08d
		M50	2.07 ± 0.07ab	2.63 ± 0.08bc	3.13 ± 0.08ab	3.41 ± 0.16b
		M100	1.90 ± 0.07abc	2.67 ± 0.01b	3.35 ± 0.15a	3.72 ± 0.02b
		M200	1.73 ± 0.11bc	2.62 ± 0.10c	3.01 ± 0.05ab	3.45 ± 0.13b
		M400	1.87 ± 0.04abc	2.10 ± 0.06d	2.76 ± 0.07b	2.94 ± 0.06c
	HN65	CK	1.98 ± 0.06a	3.04 ± 0.03a	3.36 ± 0.03a	3.88 ± 0.04a
		DS	1.17 ± 0.11 cd	2.19 ± 0.06d	2.66 ± 0.02b	3.14 ± 0.03c
		M50	2.08 ± 0.02a	2.45 ± 0.12 cd	3.18 ± 0.01a	3.52 ± 0.15b
		M100	1.69 ± 0.09b	2.87 ± 0.21ab	3.40 ± 0.13a	3.61 ± 0.01ab
		M200	1.35 ± 0.03c	2.57 ± 0.05bc	3.22 ± 0.35a	3.80 ± 0.14a
		M400	1.10 ± 0.06d	2.39 ± 0.08 cd	3.23 ± 0.07a	3.44 ± 0.04b
Petioles dry weight (g/pot)	HN44	CK	1.59 ± 0.04a	2.04 ± 0.04a	2.48 ± 0.07a	3.38 ± 0.05a
		DS	1.38 ± 0.08ab	1.74 ± 0.03b	2.09 ± 0.01c	2.47 ± 0.02 cd
		M50	1.53 ± 0.16ab	1.87 ± 0.16ab	2.23 ± 0.01bc	2.63 ± 0.11bc
		M100	1.25 ± 0.03bc	1.80 ± 0.06b	2.30 ± 0.05b	2.78 ± 0.12b
		M200	0.89 ± 0.02d	1.83 ± 0.07ab	2.10 ± 0.02c	2.50 ± 0.09bcd
		M400	0.94 ± 0.18 cd	1.39 ± 0.02c	2.08 ± 0.04c	2.33 ± 0.06d
	HN65	CK	1.30 ± 0.01a	1.73 ± 0.05a	2.05 ± 0.04a	2.33 ± 0.03a
		DS	0.90 ± 0.09c	1.15 ± 0.05d	1.56 ± 0.06d	1.83 ± 0.06d
		M50	1.23 ± 0.03b	1.52 ± 0.05 cd	1.71 ± 0.02c	2.05 ± 0.05c
		M100	1.14 ± 0.16ab	1.65 ± 0.08ab	1.86 ± 0.0b	2.09 ± 0.09bc
		M200	0.99 ± 0.03bc	1.51 ± 0.08bc	1.95 ± 0.04ab	2.28 ± 0.01ab
		M400	1.00 ± 0.04bc	1.34 ± 0.05 cd	1.89 ± 0.03b	2.11 ± 0.08bc
Root dry weight (g/pot)	HN44	CK	2.08 ± 0.05c	2.23 ± 0.03e	2.81 ± 0.01de	3.93 ± 0.32a
		DS	2.27 ± 0.01b	2.44 ± 0.02d	2.70 ± 0.10e	2.93 ± 0.06b
		M50	2.54 ± 0.01a	2.80 ± 0.06b	3.09 ± 0.03abc	3.85 ± 0.16a
		M100	2.47 ± 0.13a	3.02 ± 0.06a	3.42 ± 0.17a	3.89 ± 0.10a
		M200	2.49 ± 0.06a	2.76 ± 0.01bc	3.20 ± 0.06ab	3.70 ± 0.02a
		M400	2.45 ± 0.04ab	2.64 ± 0.03c	3.05 ± 0.14bcd	3.49 ± 0.09ab
	HN65	CK	2.24 ± 0.06b	2.81 ± 0.03a	3.27 ± 0.09a	4.18 ± 0.03a
		DS	1.80 ± 0.08d	2.10 ± 0.03c	2.41 ± 0.09c	2.80 ± 0.08e
		M50	2.04 ± 0.06c	2.38 ± 0.06b	2.78 ± 0.09b	3.01 ± 0.05d
		M100	2.55 ± 0.03a	2.88 ± 0.01a	3.12 ± 0.12a	3.49 ± 0.09b
		M200	2.52 ± 0.01a	2.98 ± 0.09a	3.22 ± 0.04a	3.62 ± 0.03b
		M400	2.69 ± 0.01a	2.85 ± 0.04a	3.01 ± 0.01ab	3.25 ± 0.03c

*Note* Refer to Table 1 for descriptions to Tables

### Effects of MC on physiological characteristics of soybean under drought stress

**Fig. 3:**
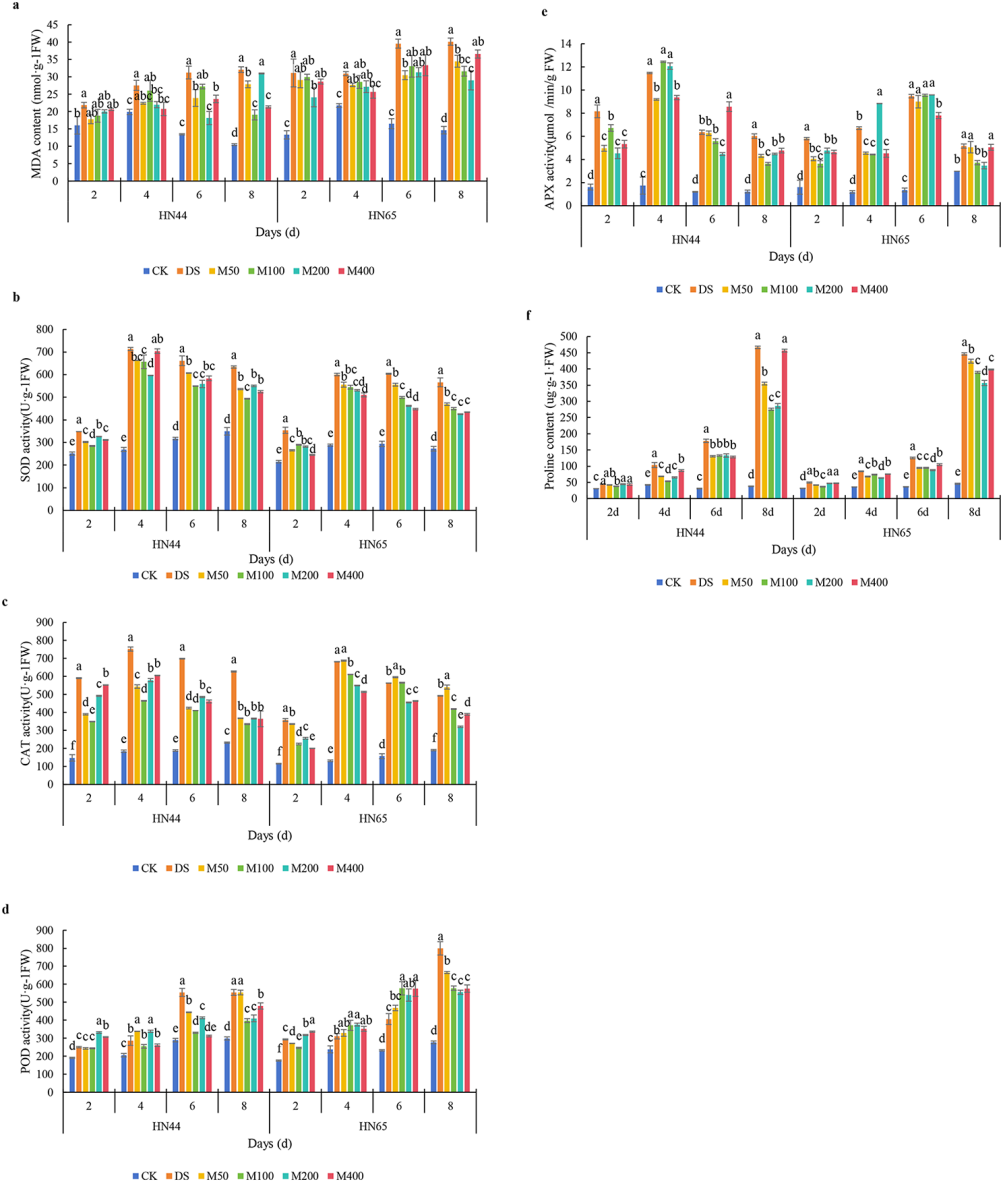
Effects of MC on physiological characteristics of soybean under drought stress. **a**, MDA content; **b**, SOD activity; **c**, CAT activity; **d**, POD activity; **e**, APX activity; **f**, proline content. *Note* Refer to Fig. 1 for descriptions to Figures

The degree of membrane lipid peroxidation reflects the extent of the cellular damage. With prolonged drought stress, the malondialdehyde content in the two varieties gradually increased, reaching a peak on day 8, and the content of the sensitive variety Heinong 65 was higher. The application of MC relatively alleviated the accumulation of malondialdehyde under drought stress; however, it remained higher than that in the CK treatment (Fig. 3a). In Heinong 44, each MC treatment reduced the malondialdehyde content under drought stress. On day 8, the decrease in MDA for each treatment was in the order of M100 > M400 > M50 > M200. Compared with the DS treatment, the content of malondialdehyde in the M100 treatment decreased by 14.03%, 5.55%, 12.90%, and 40.60%, respectively. In Heinong 65, all MC treatments reduced malondialdehyde content under drought stress on all days except for the M200 treatment, which was higher on day 2. On day 8, the decrease in MDA for each treatment was in the order of M200 > M100 > M50 > M400. Compared with DS, the content of malondialdehyde in the M200 treatment decreased by 22.51%, 12.13%, 20.82%, and 27.75%, respectively.

The SOD activity first increased and then decreased. The drought-resistant variety Heinong 44 reached its highest value on day 4, and the sensitive variety Heinong 65 reached its highest value on day 6. The application of MC relatively alleviated the increase in SOD activity under drought stress (Fig. 3b). In Heinong 44, MC reduced SOD activity under drought stress. On day 8, the decrease in SOD activity in each treatment was in the order of M100 > M50 > M400 > M200. Compared with DS, the SOD activity of M100 treatment decreased by 18.24%, 7.90%, 16.95%, and 22.11%, respectively. In Heinong 65, the decrease in SOD activity in each treatment group on day 8 was in the order of M200 > M400 > M100 > M50. Compared with DS, the SOD activity of M200 treatment decreased by 20.43%, 11.60%, 23.51%, and 24.83%, respectively.

CAT activity first increased and then decreased with the prolongation of drought stress time, reaching the highest value on day 4 (Fig. 3c). In Heinong 44, all MC treatments reduced CAT activity under drought stress. On day 8, the decrease in CAT for each treatment was in the order of M100 > M50 > M200 > M400. Compared with the DS treatment, the CAT activity of the M100 treatment decreased by 40.86%, 38.23%, 41.21%, and 46.59%, respectively. In Heinong 65, the decrease in CAT activity for each treatment on day 8 was in the order M200 > M400 > M100 > M50. Compared with the DS treatment, the CAT activity of the M200 treatment decreased by 28.35%, 19.39%, 19.10%, and 34.90%, respectively.

POD activity increased gradually and reached its highest value on day 8. The application of MC alleviated the increase in POD activity under drought stress to a certain extent; however, it remained higher than that of the CK treatment (Fig. 3d). The POD activity for each MC treatment generally showed an increasing trend. In Heinong 44, all MC treatments reduced POD activity under drought stress after day 6. On day 8, the decrease in POD for each treatment was in the order M100 > D20 > M400 > M50. Compared with the DS treatment, POD activity in the M100 treatment decreased by 2.59%, 11.06%, 40.42%, and 28.25%, respectively. In Heinong 65, all MC treatments reduced POD activity under drought stress on day 8. On day 8, the decrease in POD for each treatment was in the order M200 > M400 > M100 > M50. Compared with DS treatment, the POD activity of M200 treatment decreased by − 8.27%, − 20.87%, − 32.84%, and 30.52%, respectively. Under drought stress, the POD activity of the two types generally decreased with varying doses of MC. POD became increasingly crucial for drought resistance as the drought duration increased.

The APX activity first increased and then decreased (Fig. 3e). The drought-resistant variety Heinong 44 reached its highest value on day 4, and the sensitive variety Heinong 65 reached its highest value on day 6. The application of MC alleviated the increase in APX activity under drought stress to a certain extent; however, it remained higher than that in the CK treatment. In Heinong 44, all MC treatments reduced APX activity under drought stress, except on day 4. On day 8, the decrease in APX for each treatment was in the order of M100 > M50 > M200 > M400. Compared to DS, the APX activity of M100 treatment decreased by 17.62%, -8.48%, 12.05%, and 39.76%, respectively. In Heinong 65, the decrease in APX for each treatment on day 8 was in the order M200 > D1400 > M400 > M50. Compared with DS, the APX activity of the M200 treatment decreased by 17.76%, -31.70%, -1.09%, and 32.70%, respectively.

The proline content of the two varieties in the CK treatment was maintained at a low level. With prolonged drought stress, the proline content in the two varieties gradually increased, reaching a peak on day 8. MC relatively alleviated proline accumulation under drought stress conditions (Fig. 3f). In Heinong 44, each MC treatment reduced proline content under drought stress. On day 8, the proline content decreased in the following order: M100 > M400 > M500 > M200. Compared with DS, the proline content in the M100 treatment decreased by 15.41%, 48.39%, 25.47%, and 40.94%, respectively. In Heinong 65, each MC treatment reduced proline content under drought stress. On day 8, proline content decreased in the following order: M200 > M100 > M50 > D40. Compared to DS, the proline content in the M200 treatment decreased by 5.37%, 24.44%, 30.09%, and 20.07%, respectively.

Generally, each MC treatment affected the physiological parameters of both varieties. The effect of an MC concentration of 100 and 200 mg/L on Heinong 44 and Heinong 65, respectively, was better.

### MC alleviated the photosynthetic damage caused by drought stress

**Fig. 4:**
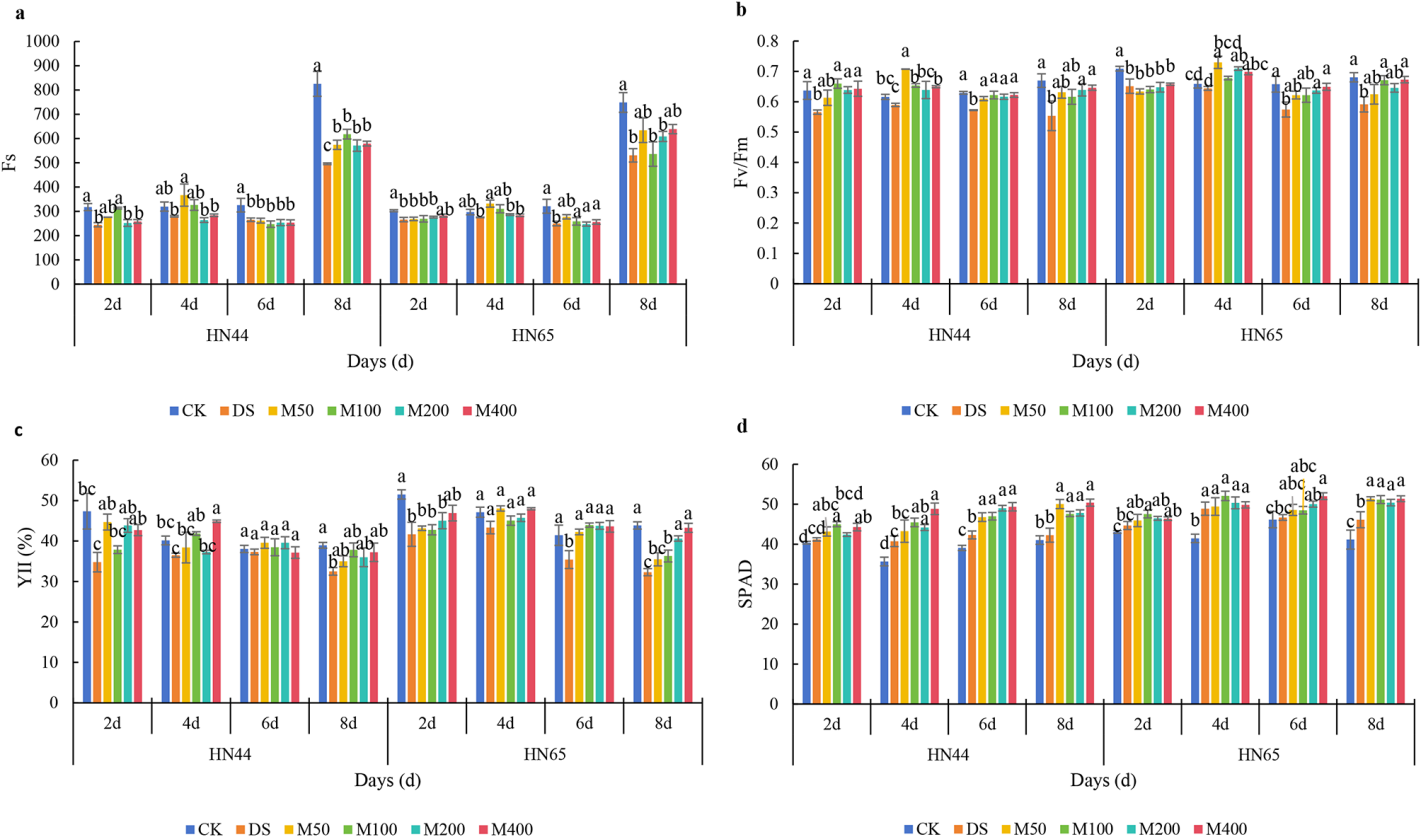
Effects of MC on chlorophyll fluorescence parameters under drought stress. **a**, Fs; **b**, Fv/Fm; **c**, YII; **d**, SPAD index. *Note* Refer to Figure 1 for descriptions to Figures

Drought stress led to a decrease in Fs, and with the extension of drought stress time, Fs was more strongly inhibited (Fig. 4a). In Heinong 44, drought caused Fs to decrease by 22.97%, 12.40%, 18.47%, and 39.92% on days 2, 4, 6, and 8, respectively. In Heinong 65, drought caused Fs to decrease by 12.03%, 7.37%, 22.14%, and 29.09%, respectively. The application of MC relatively alleviated the decrease in Fs under drought stress; however, the effect was weak during the early stages of the experiment. Over time, the mitigation effects increased. In Heinong 44, the improvement in Fs for each treatment on day 8 was in the order of M100 > M400 > M50 > M200. Compared with DS, Fs of the M100 treatment increased by 28.12%, 16.51%, -6.83%, and 24.61%, respectively. In Heinong 65, the increase in Fs for each treatment on day 8 was in the order M50 > M400 > M200 > M100. Compared to DS, the Fs of the M50 treatment increased by 1.03%, 20.64%, 10.94%, and 19.48%, respectively.

Figure 4b shows the changes in PSII photochemical quantum efficiency (Fv/Fm) in each treatment during the test period; drought stress led to a significant decrease in Fv/Fm. In Heinong 44, a significant difference was observed between the DS and CK treatments on days 4 and 8. Drought caused the Fv/Fm to decrease by 11.17%, 4.30%, 9.09%, and 17.35%, respectively. In Heinong 65, the DS and CK treatments were significantly different each day, and drought caused Fv/Fm to decrease by 8.15%, 2.23%, 12.71%, and 13.08%, respectively. The application of MC alleviated the decrease in Fv/Fm under drought stress to a certain extent. In Heinong 44, each MC treatment was higher than the DS treatment daily. On day 8, the effect of each treatment on Fv/Fm was in the order of M400 > M200 > M100 > M50, and there was no significant difference between the treatments. Compared with DS, Fv/Fm in the M400 treatment increased by 13.61%, 10.19%, 8.72%, and 16.75%, respectively. In Heinong 65, different concentrations of MC affected Fv/Fm, and there was a significant difference between the M400 and DS treatment on days 6 and 8. On day 8, the improvement in Fv/Fm for each treatment was in the order of M400 > M200 > M50 > M100. Compared to DS, the Fv/Fm of the M400 treatment increased by 1.01%, 8.43%, 13.06%, and 13.76%, respectively.

Drought stress significantly reduced YII, inhibited the actual light energy conversion efficiency, and caused photosynthetic damage (Fig. 4c). In Heinong 44, on days 2, 4, 6, and 8, the DS treatment was lower than the CK treatment, and drought caused qL to decrease by 26.58%, 9.09%, 1.92%, and 16.56%, respectively. In Heinong 65, the YII decreased gradually during the test and reached its lowest value on day 8. Drought caused YII to decrease by 19.12%, 8.06%, 14.53%, and 26.34%, respectively. The application of MC alleviated the decrease in YII under drought stress to ensure that the plants could maintain a certain photosynthetic process under drought stress. In Heinong 44, different concentrations of MC alleviated the decrease in the YII. On day 8, the YII value of the M100 treatment was greater than that of the CK treatment, and the improvement effect of each treatment on qL was in the order of M100 > M400 > M200 > M50. Compared to DS, YII in M100 treatment increased by 8.86%, 14.66%, 3.05%, and 16.37%, respectively. In Heinong 65, different concentrations of MC had a greater effect on YII improvement. Except for the M200 treatment on day 2, which was lower than the DS treatment, each treatment was higher than the DS treatment on the other days, and on day 8, it was significantly different from the DS treatment. On day 8, the improvement in qL for each treatment was in the order of M400 > M200 > M100 > M50. Compared to DS, qL of M100 treatment increased by 12.60%, 10.71%, 23.15%, and 33.96%, respectively.

Figure 4d shows the SPAD changes for each treatment during the test period. Drought stress promoted an increase in SPAD. In Heinong 44, drought increased SPAD by 2.04%, 14.25%, 8.31%, and 2.99% on days 2, 4, 6, and 8, respectively. In Heinong 65, drought increased SPAD by 3.70%, 17.93%, 1.11%, and 12.06%, respectively. The application of MC further increased SPAD content under drought stress. In Heinong 44, different concentrations of MC increased the SPAD values. On day 8, the difference between each treatment and the DS treatment was significant, and the effect of each treatment on SPAD was in the order M400 > M50 > M200 > M100. Compared to DS, the SPAD of the M400 treatment increased by 7.49%, 19.91%, 16.67%, and 19.25%, respectively. In Heinong 65, different concentrations of MC increased the SPAD values. On day 8, a significant difference was observed between each treatment and the DS treatment, and the improvement in SPAD by each treatment was in the order M50 > M400 > M200 > D10. Compared to DS, the SPAD of the M50 treatment increased by 2.82%, 1.09%, 4.05%, and 11.41%, respectively.

Generally, MC increased chlorophyll fluorescence parameters under drought stress and alleviated the photosynthetic damage caused by drought. Different concentrations of MC showed better mitigation effects on Heinong 44 and 65.

## Discussion

### MC has a regulatory effect on plant type and yield traits

MC is the most widely used in cotton and is extensively researched. By preventing apical dominance, MC application compacts the cotton plant type and influences the production, quality, and spatial distribution of cotton bolls in the field [16]. In addition, MC induces several GA catabolism genes, including *GA2ox*, resulting in a decrease in plant height [17]. In soybeans, although MC induces a decrease in plant height, it does not entirely depend on the GA pathway. In our previous study, differentially expressed genes related to cell-wall synthesis and plant hormone signal transduction pathways were significantly downregulated at the transcriptional level [18]. Combined with the results of the present study, the number of nodes and stem lengths of the bottom and middle internodes of the two varieties decreased. The number of internode of Heinong 44 under M100 and M400 treatments and Heinong 65 under M50, M200, M400 and M800 treatments was significantly different from that of CK (Table 2). Li et al. [19] found that the reduction of soybean nodes was beneficial to the increase of yield under dense planting conditions, which was consistent with the results of this study. This indicates that MC-induced plant height reduction in soybeans is achieved by inhibiting the number and length of internodes.

MC can also regulate yield and can improve the generation and use efficiency of carbohydrates in leaves, delay the onset of leaf senescence, and increase cotton output. In the field, chlorophyll content, starch, and non-structural carbohydrate productivity increased, whereas carbon mobilization, non-structural carbohydrates, and seed cotton yield were positively correlated [20, 21]. In addition, another perspective suggests that MC is usually closely related to planting density and does not directly affect yield formation but improves light-use efficiency and yield by improving plant structure at high plant densities [22]. According to the results of this study, experiments with many varieties for many years showed that MC promotes soybean yield. In terms of yield characteristics, some differences exist among different varieties; however, the number of two-grain or three-grain pods usually increases, and the yield-increasing effect differs in different years, which may be related to the water conditions of the year. Natural precipitation during the application period may affect the MC to a certain extent. Another study on soybeans showed that MC could improve the source-sink relationship and yield of soybeans, which manifested as an increase in dry matter accumulation/plant, pod number, 100-seed weight, and seed yield [23]. However, in the present study, the effect of MC on the 100-seed weight was limited to varieties, and there were some differences among different varieties, which increased or decreased at different concentrations.

In soybeans, the application of MC relatively increased the dry weight of the aboveground and underground parts, but the concentration of MC was selective among the different varieties. For HN44, although different concentrations of MC increased the yield under field conditions, the yield began to decline when the concentration of MC exceeded 400 mg/L. Under drought conditions, the 400 mg/L concentration of MC treatment aggravated the dry weight loss of leaves. Similarly, in the field experiment over two consecutive years, the yield of HN84 at a concentration of 400 mg/L was lower than that of the M200 treatment.

### MC improved the physiological characteristics of soybean under field conditions

The effects of MC on the physiological characteristics of soybeans under field conditions have not yet been reported. In the present study, MC improved many physiological characteristics under field conditions, including antioxidant properties and proline content. From R2 to R6, the MC played a role in each growth period. SOD activity showed a single-peak curve and peaked at the R4 stage. POD activity continued to increase and reached a peak at the R6 stage. The upregulation of these antioxidant enzymes at different stages can potentially resist the water-deficient environment in the field without affecting the yield. Proline gradually accumulated in the two varieties, reaching a peak at the R6 stage, and the accumulation of proline also contributed to an improvement in stress resistance [24]. Currently, no study has clarified the direct relationship between proline and yield; however, proline may be beneficial for yield. Studies have shown that proline can alleviate the adverse effects of drought and increase strawberry yield in water-deficient environments. The use of exogenous proline has significantly increased strawberry berry yield by 23% [25]. Importantly, the relative chlorophyll content also increased (Fig. 1d ). At the R5 stage, the difference between each treatment and CK was significant, which may be more conducive to photosynthesis by enhancing the function of the source to increase the accumulation of the sink, thereby affecting the yield. However, the application of MC may be limited by many factors, including species, application period, and experimental site. A study conducted in the Yangtze River Basin in China revealed that MC application decreased photosynthesis and resulted in a decrease in yield by increasing the accumulation of sucrose, hexose, and starch in late-sown cotton leaves [26].

### Regulation effect of MC on drought stress

The physiological effects of MC on crops in the field indicate its potential application for stress resistance. In the study of Shahrbano et al. [27], MC treatment of cotton before drought could increase root dry weight by 28% and leaf dry weight by 34%. The application of MC increased the dry weight of each part of soybean under drought stress. When the effect was the best, the dry weight of leaves, stems, petioles, and roots increased by 38.42%, 49.40%, 24.59% and 41.90%. Therefore, the use of MC can effectively improve the damage caused by drought stress to plant growth, but it is necessary to pay attention to the concentration of MC, and too high concentration will further inhibit plant growth.

MC application increases the net photosynthetic rate of cotton leaves and triggers the formation of carbohydrates by improving the source-sink relationship [10]. Han et al. [28] found that the application of MC effectively increased the root activity, POD activity, and chlorophyll content of tall fescue seedlings. In contrast, Du et al. [29] found that MC improved the peroxidase (POD) activity of sweet potato roots and reduced their malondialdehyde (MDA) content under drought stress conditions. According to our previous study, MC notably lowered soybean plant height and shoot dry weight, promoted the development of lateral roots, boosted the activity of protective enzymes in leaves, decreased the amount of MDA, and increased the amount of total flavonoids [30]. Therefore, foliar application of MC is helpful to improve drought resistance.

According to Wang et al. [31], MC improves energy efficiency during salt stress by upregulating ATP synthase and downregulating cytochrome b5-like reductase, which improves ATP synthesis and decreases the generation of ROS. Under drought stress, MC effectively alleviated oxidative damage in soybeans, which was intuitively manifested as a significant decrease in MDA (Fig. 3a). MDA, a lipid peroxidation product formed by the lipid peroxidation of ROS with phospholipids, enzymes, and membrane receptors related to polyunsaturated fatty acids and nucleic acids, reflects a decrease in ROS levels in plants to a certain extent. Therefore, compared with the DS treatment, antioxidant enzyme activity and proline content decreased but remained higher than those in the CK treatment (Fig. 3). In another study, we found that MC promoted the accumulation of some amino acids and flavonoids to withstand drought at the metabolic level [32]. Lee et al. [33] reported that MC pretreatment before drought promoted drought resistance in cotton during the peak water demand period and reduced the risk of drought. The MC treatment produced a larger seed surface area, seed boll number, and boll quality. In soybeans, the application of MC relatively increased the dry weight of the aboveground and underground parts, but the concentration of MC was selective among the different varieties. For HN44, although different concentrations of MC increased the yield under field conditions, the yield began to decline when the concentration of MC exceeded 400 mg/L. Under drought conditions, the 400 mg/L concentration of MC treatment aggravated the dry weight loss of leaves. Similarly, in the field experiment over two consecutive years, the yield of HN84 at a concentration of 400 mg/L was lower than that of the M200 treatment.

MC also affected photosynthesis, increased the SPAD index under field and drought conditions, and improved chlorophyll fluorescence parameters. The application of DPC also affects photosynthesis in plants and changes the distribution of photosynthates and the absorption and fixation of CO_2_ [26]. For the R2–R6 periods, under field conditions, different concentrations of MC increased the SPAD index; the closer it was to the R6 period, the higher the SPAD value and the more conducive it was for increased yield (Fig. 1d). Gupta et al. [34] found that, after foliar spraying with MC, millet plant height decreased, chlorophyll content and relative water content increased significantly, specific leaf weight increased significantly, and grain yield increased significantly. Under drought stress, Fs, Fv/Fm, YII, and SPAD increased with increasing MC. The improvement in the actual light energy conversion efficiency was conducive to the smooth progress of photosynthesis and the synthesis of more energy to resist drought. Dos Santos et al. [35] found that MC enhanced the physiological systems of Eucalyptus plants, enhanced their intrinsic water usage efficiency and SPAD index, and decreased the stomatal conductance and transpiration rate. Furthermore, it led to a decrease in tree height but an increase in root dry weight, total dry weight, leaf number, and secondary branch number, all of which supported the drought resilience of Eucalyptus clones. Gao et al. [36] found that spraying different concentrations of MC at different growth stages increased the leaf area index, Pn, and chlorophyll content. In summary, MC has great application potential in field production and drought stress. Therefore, it is possible to find an equilibrium level that can improve drought resistance as well as ensure no reduction in yield. Spraying 100–200 mg/L of MC in the field has the best effect and reduces the cost of chemicals.

## Conclusion

In conclusion, we demonstrated the role of MC in regulating soybean yield and drought resistance through field and drought experiments. The application of MC improved the yield formation characteristics of soybeans as well as regulated the plant height, effectively reduced the MDA level, and improved the photosynthetic characteristics. However, soybeans are selective to MC concentrations, and it is difficult to achieve a balance between yield and drought resistance at high concentrations. When the MC level was maintained at 100–200 mg/L, the yield and resistance effectively increased. This study has significant theoretical and practical implications and offers a theoretical justification for the sensible implementation of MC in soybeans. However, the current research is not comprehensive, and lacks intuitive photosynthetic parameters such as transpiration rate and stomatal conductance, so it is difficult to understand the effect of MC on photosynthesis. Moreover, there are still many gaps in the molecular mechanism of MC for soybean plant type or yield formation. In the future, we will further carry out more in-depth research.

### Electronic supplementary material

Below is the link to the electronic supplementary material.

Supplementary Material 1

## Data Availability

Data is provided within the manuscript or supplementary information files.

## Declarations

### Supplementary Information

Table S1 Soil basic fertility.
